# Supplementary material for: Fast and powerful genome wide association of dense genetic data with high dimensional imaging phenotypes
Source: Nat Commun. 2018 Aug 14;9:3254. doi: 10.1038/s41467-018-05444-6 (PMC6092439; doi:10.1038/s41467-018-05444-6)
Supplement: Supplementary file 1 — Supplementary Information [file 41467_2018_5444_MOESM1_ESM.pdf]

Supplementary Information for ‘Fast and Powerful Genome Wide Association Analysis of Dense Genetic Data with High Dimensional Imaging Phenotypes’

Ganjgahi et.al

Supplementary Note 1

Intensive simulation studies are conducted to evaluate proposed methods for association estimation and testing. The aim of the first study is to compare fully converged and one-step random effect estimators based on the simplified ML and REML functions. In the second study, the performance of various test statistics for the association testing are compared using a fully converged or one-step random effect estimators for ML and REML functions. Finally, we compare FaST-LMM [Lippert et al., 2011] to our preferred test, the score test based on the simplified REML function, $\mathbf{T}_{\mathrm{S,REML}}$, using both false positive error rates and empirical power using simulated genetic markers.

In all simulations the response variable is assumed to follow $Y = X\beta + \epsilon$, where $\epsilon \sim N(0, \Sigma)$ and $\Sigma = \sigma^2_{\mathrm{A}}(2\Phi) + (1 - \sigma^2_{\mathrm{A}})I$, giving a unit variance phenotype. As above, the design matrix is partitioned $X = [X_1 X_2]$, where X_1 is the allele count per subject for a given marker, and X_2 are all other non-genetic fixed effects. In our simulations, X_1 is based on simulated marker, where each marker has a reference allele frequency sampled from a uniform distribution on $[0.1, 0.9]$. The X_2 matrix has 3 columns, an intercept, a linear trend from -1 to 1, and the element-wise square of the linear trend. Kinship matrices from a family study, genetic analysis workshop 10 (GAW10), and genetic similarity matrix from simulated genetic markers for a sample of unrelated individuals with different sizes were chosen to set the covariance, for a range of genetic variances, $\sigma^2_{\mathrm{A}} = 0, 0.2, 0.4, 0.6\&0.8$. Specifically, the Cholesky decomposition of Σ was used to premultiply i.i.d normal random variables with 5000 realisations.

Supplementary Note 2

FaST-LMM and the score test performances based on P-value, parameter estimate $(\hat{\beta}_1)$ and variance of parameter estimate $(\mathrm{var}(\hat{\beta}_a))$ are compared using simulated SNPs and phenotype when there is no population structure. 60,000 SNPs for 300 individuals with minor allele frequency between $(0.05, 0.5)$ were simulated. 6000 null and 100 causal markers were used to compare the false positive rates in 5000 realisations. In the null simulations 54000 markers were used to induce different level of heritability under the additive model $h^2_{\mathrm{A}} = 0.1, 0.2, 0.4, 0.6\&0.8$ and $\sigma^2_p = 1$ where markers are standardised to have mean zero and unit variance. Finally for power simulations 100 markers were explained 30% of phenotipic variance where the effect for each marker was drawn from $N(0, 0.3/100)$.

The final simulation study evaluates controlling for a heritable fixed effect nuisance covariate in the null simulation setting when there is neither a SNP effect nor a covariate effect on the phenotype. Although the LMM can accommodate fixed effect nuisance terms, we compare to an alternate approach where nuisance covariates are regressed out in advance and LMM is fitted to the residualized phenotypes for GWA. We note that imaging association studies routinely use intracranial volume (ICV) as a nuisance covariate [Hibar et al., 2015, Stein et al., 2012], and ICV is well known to have heritability as large as 0.8 [Peper et al., 2007, Glahn et al., 007]. 60,000 SNPs for 4,000 individuals with minor allele frequency between $(0.05, 0.5)$ were simulated when there is no population structure. In the null simulations 54000 markers were used to induce different level of heritability under the additive model $h^2_{\mathrm{A}} = 0.1, 0.2, 0.4, 0.6\&0.8$ and $\sigma^2_p = 1$ where markers are standardised to have mean zero and unit variance. Additional 10 null SNPs were used to simulate nuisance heritable covariate $(h^2 = 0.6)$ under the additive model [Widmer et al., 2014] and comparing when there is no population structure FaST-LMM, EMMAX and score test based on simplified REML function using Non-iterative random effect estimator (NINGA) in terms of null distribution of parametric P-values.

Supplementary Figures

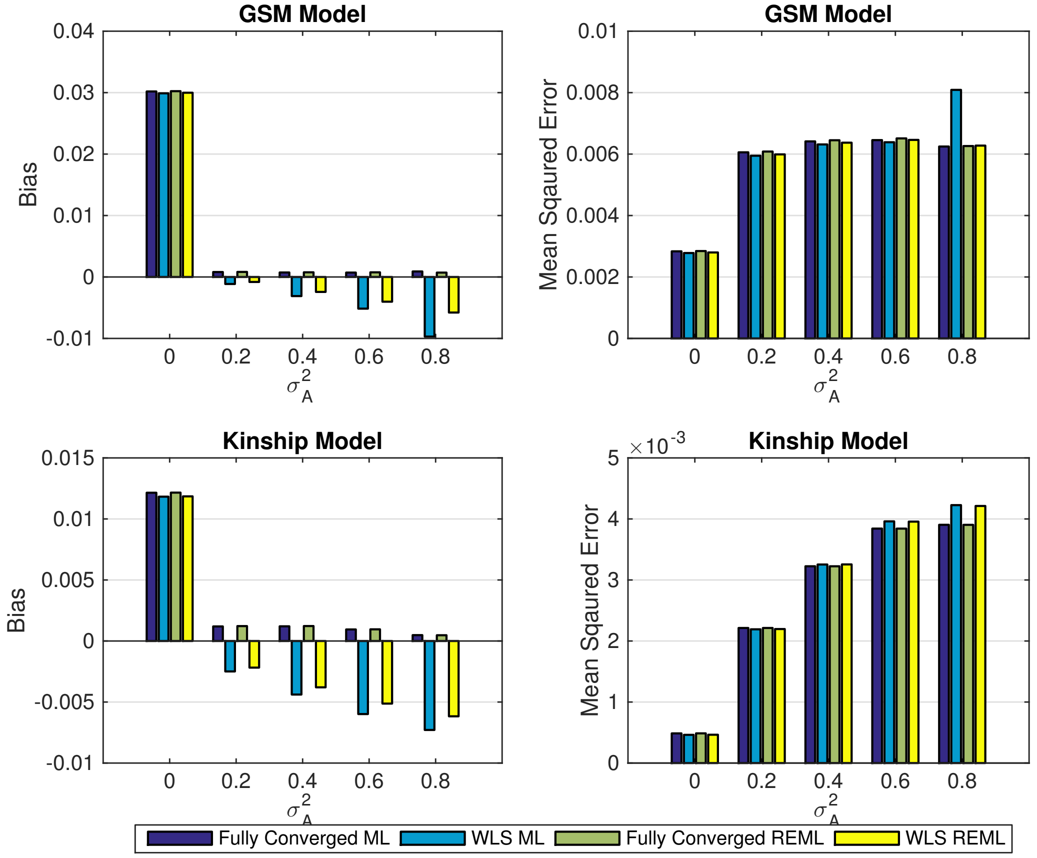

Supplementary Figure 1: Simulation 1 results, comparing the bias (left column) and mean squared error (right column) of non-iterative and fully converged random effect estimators using the simplified ML or REML for 5000 realizations, for different level of genetic random effect σ^2_{A}. The results are based on a GRM constructed from 1200 unrelated individuals (top row) and kinship matrix from GAW 10 with 23 families and 1497 individuals (bottom row). While the one-step estimators generally (ML or REML) have more bias than fully converged ones, WLS-REML has less bias than WLS-ML, and in terms of MSE there is a relatively small difference in performance among all the methods.

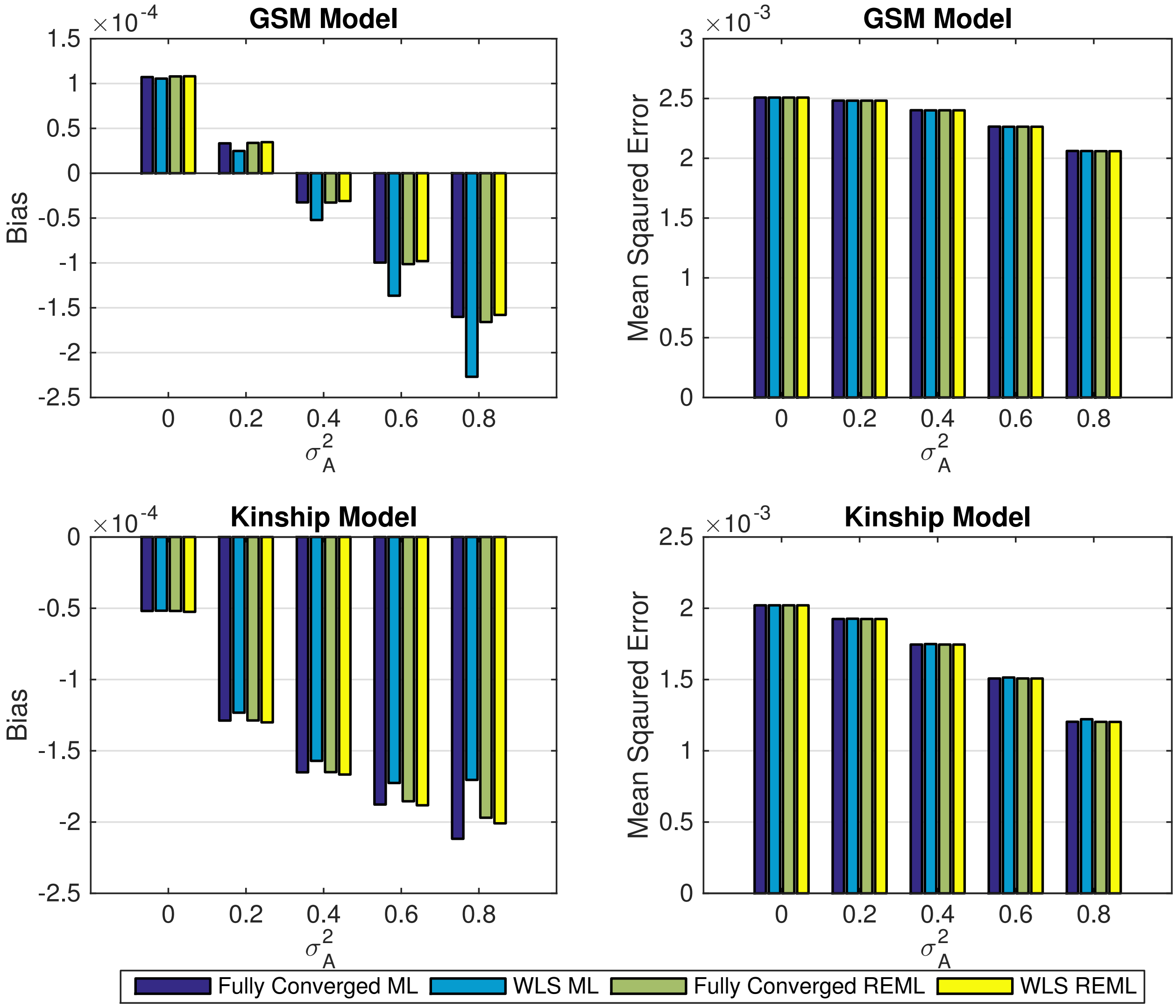

Supplementary Figure 2: Simulation 1, comparing the bias (left column) and mean squared error (right column) of the fixed effect (additive allelic effect, β_1) using the simplified ML or REML for 5000 realizations, for different level of genetic random effect σ^2_{A} when $\beta_1 = 0$. The results are based on a GRM constructed from 1200 unrelated individuals (top row) and kinship matrix from GAW 10 with 23 families and 1497 individuals (bottom row). These results show that fixed effect estimation using WLS-REML variance component estimator has almost identical performance as the fully converged one over different levels of genetic variance.

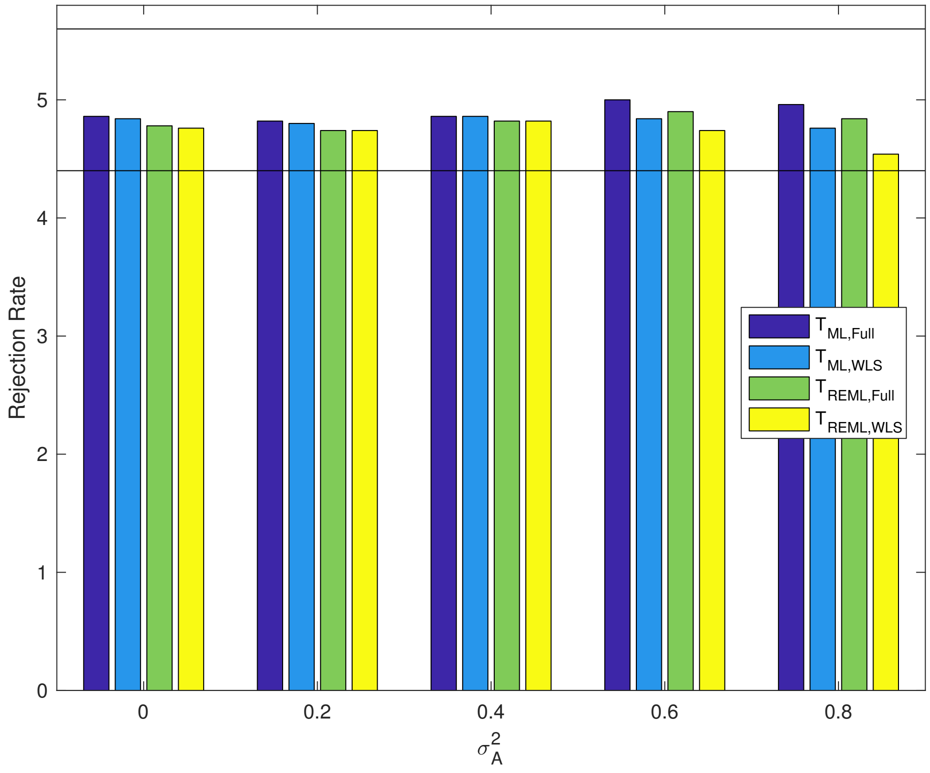

(a) Parametric error rate comparisons

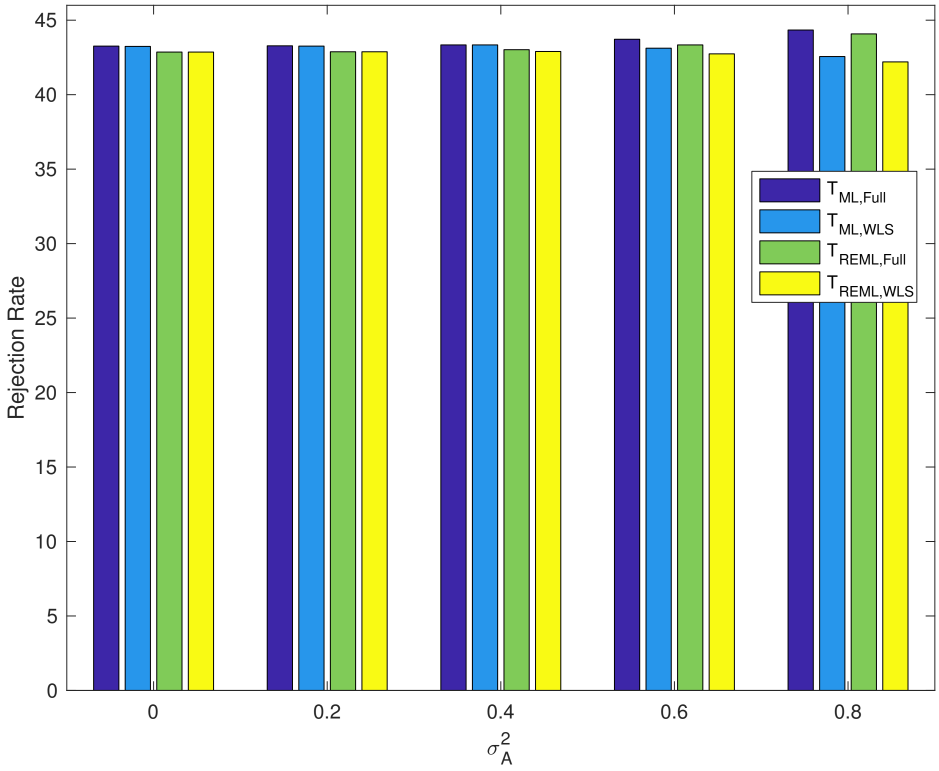

(b) Parametric power comparisons

Supplementary Figure 3: Simulation 2, comparing the simplified ML and REML score test parametric rejection rates using the one-step and the fully converged random effect estimator, 5% nominal (a) and power (b) based on simulation using either a GRM from 300 unrelated individuals or a kinship from GOBS study with 171 individuals and 10 families and 5000 realizations. Monte Carlo confidence interval is (4.40%, 5.60%). Regardless of kinship matrix in the simulation and variance component estimator, non-iterative or fully converged, all methods have similar performances.

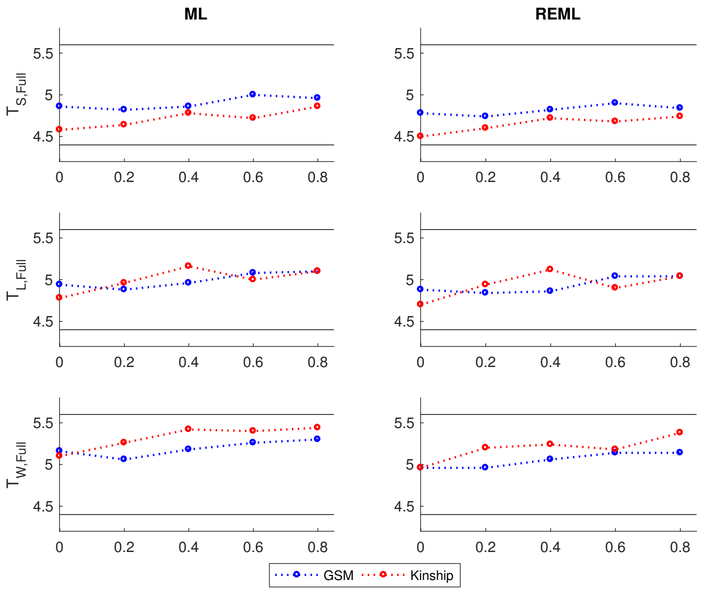

(a) Fully converged random effect estimator

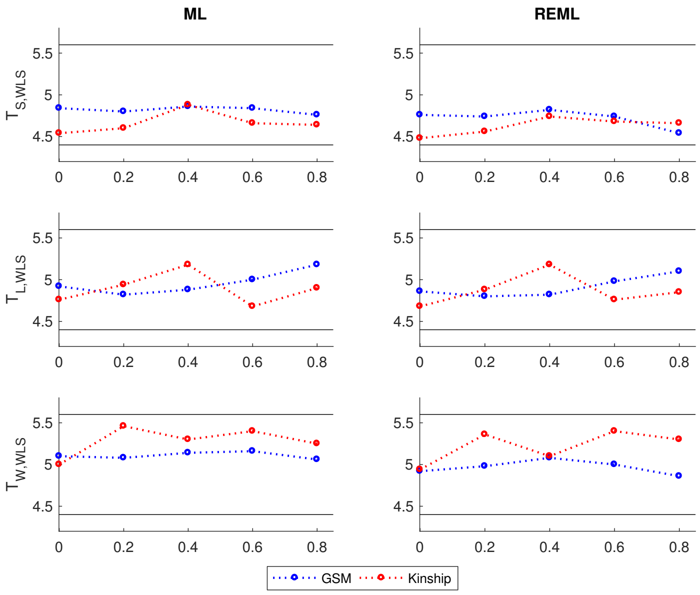

(b) one-step random effect estimator

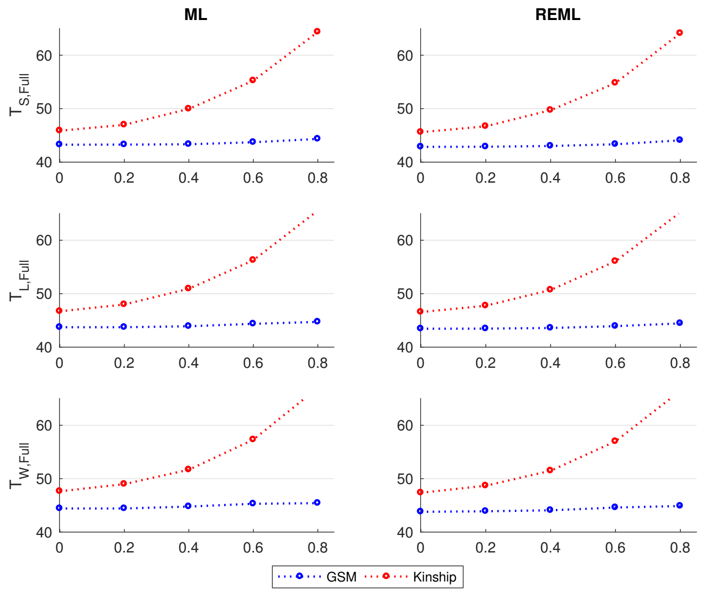

(c) Fully converged random effect estimator

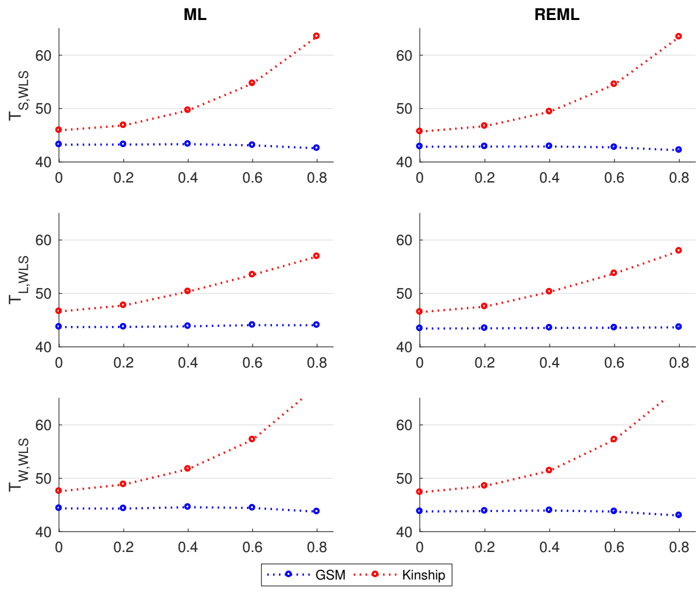

(d) one-step random effect estimator

Supplementary Figure 4: Simulation 2, comparing proposed statistics parametric error rates, 5% nominal (Top panels) and power (bottom panels) based on simulation using either a GRM from 300 unrelated individuals or a kinship from GOBS study with 171 individuals and 10 families and 5000 realisations. The panels (a) and (c) correspond to association statistics using the fully converged random effect estimator and (b) and (d) show the result using the non-iterative random effect estimator. Monte Carlo confidence interval is (4.40%, 5.60%). Regardless of kinship matrix in the simulation and variance component estimator, non-iterative or fully converged, all statistic have similar performances.

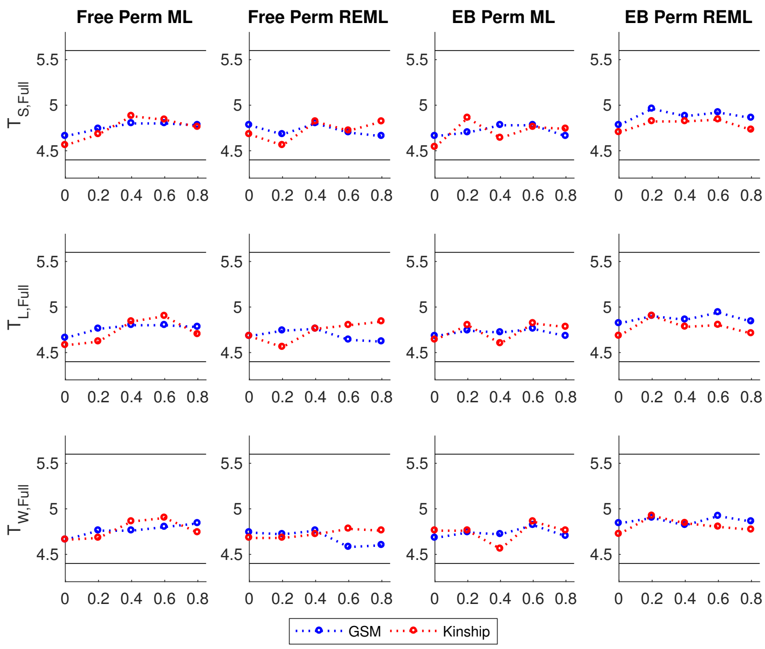

(a) Fully Converged random effect estimator

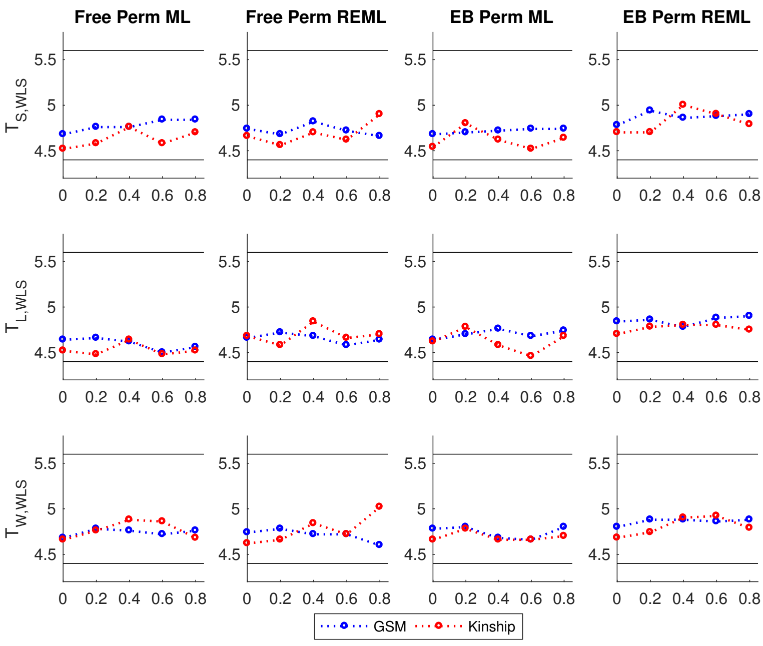

(b) one-step random effect estimator

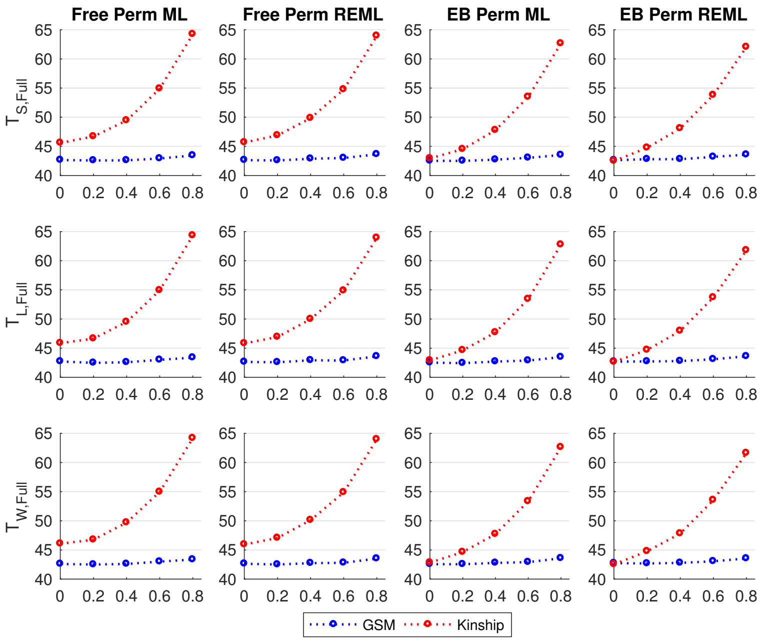

(c) Fully converged random effect estimator

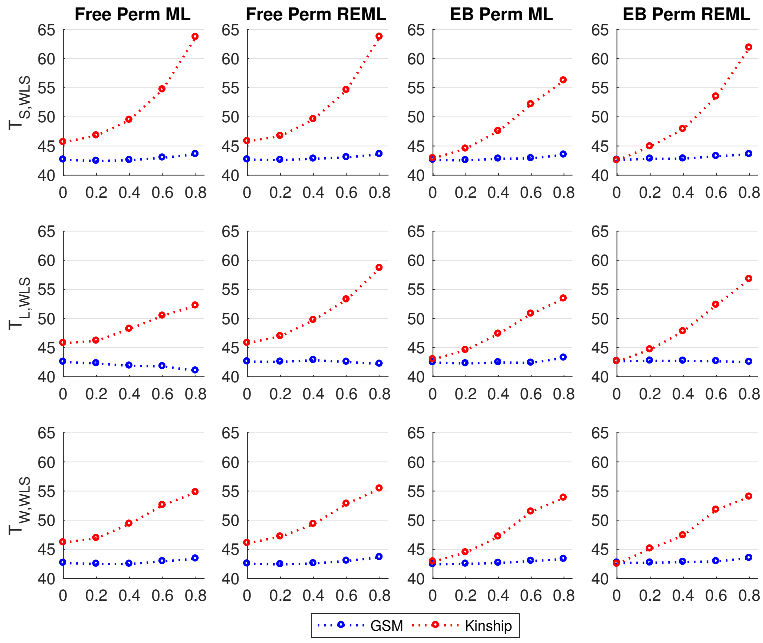

(d) one-step random effect estimator

Supplementary Figure 5: Simulation 2, comparing proposed statistics permutation based error rates, 5% nominal (Top panels) and power (Bottom panels) based on simulation using either a GRM from 300 unrelated individuals or a kinship from GOBS study with 171 individuals and 10 families and 5000 realisations and 500 permutations each realisations. The panels (a) and (c) correspond to association statistics using the fully converged random effect estimator and (b) and (d) show the result using the non-iterative random effect estimator. Monte Carlo confidence interval is (4.40%, 5.60%). Despite the kinship matrix in the simulation and variance component estimator, non-iterative or fully converged, all statistic have similar performances. Both permutation schemes could control the error rate at the nominal level, however free permutation is slightly more powerful than the restricted permutation.

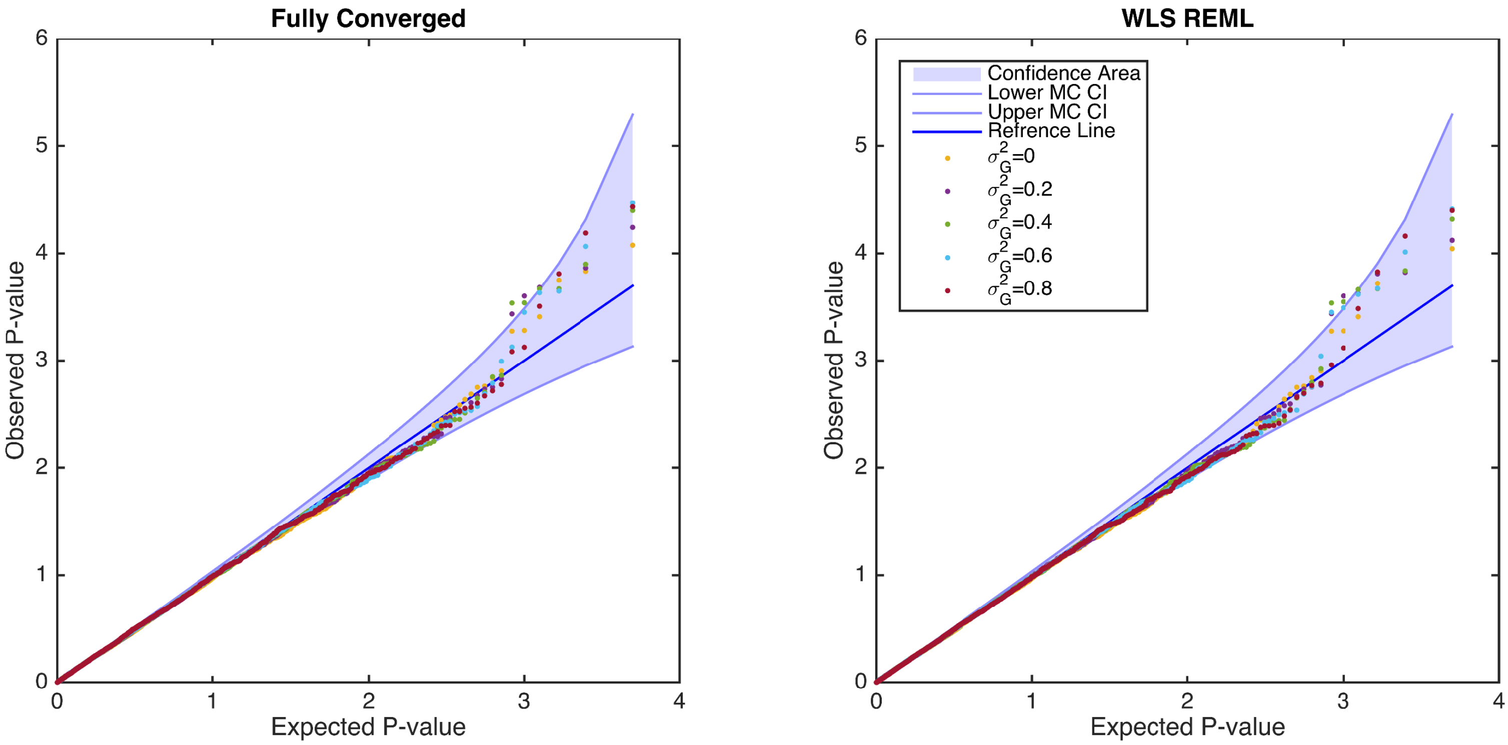

Supplementary Figure 6: Simulation 3, comparing score statistic parametric null distribution for $H_0 : \beta_1 = 0$ derived from the simplified REML function using non-iterative and fully converged random effect estimator, for a kinship from GOBS study using 10 families and 171 individuals . There is no apparent difference between the two random effect estimators, and both are consistent with a valid (uniform) P-value distribution.

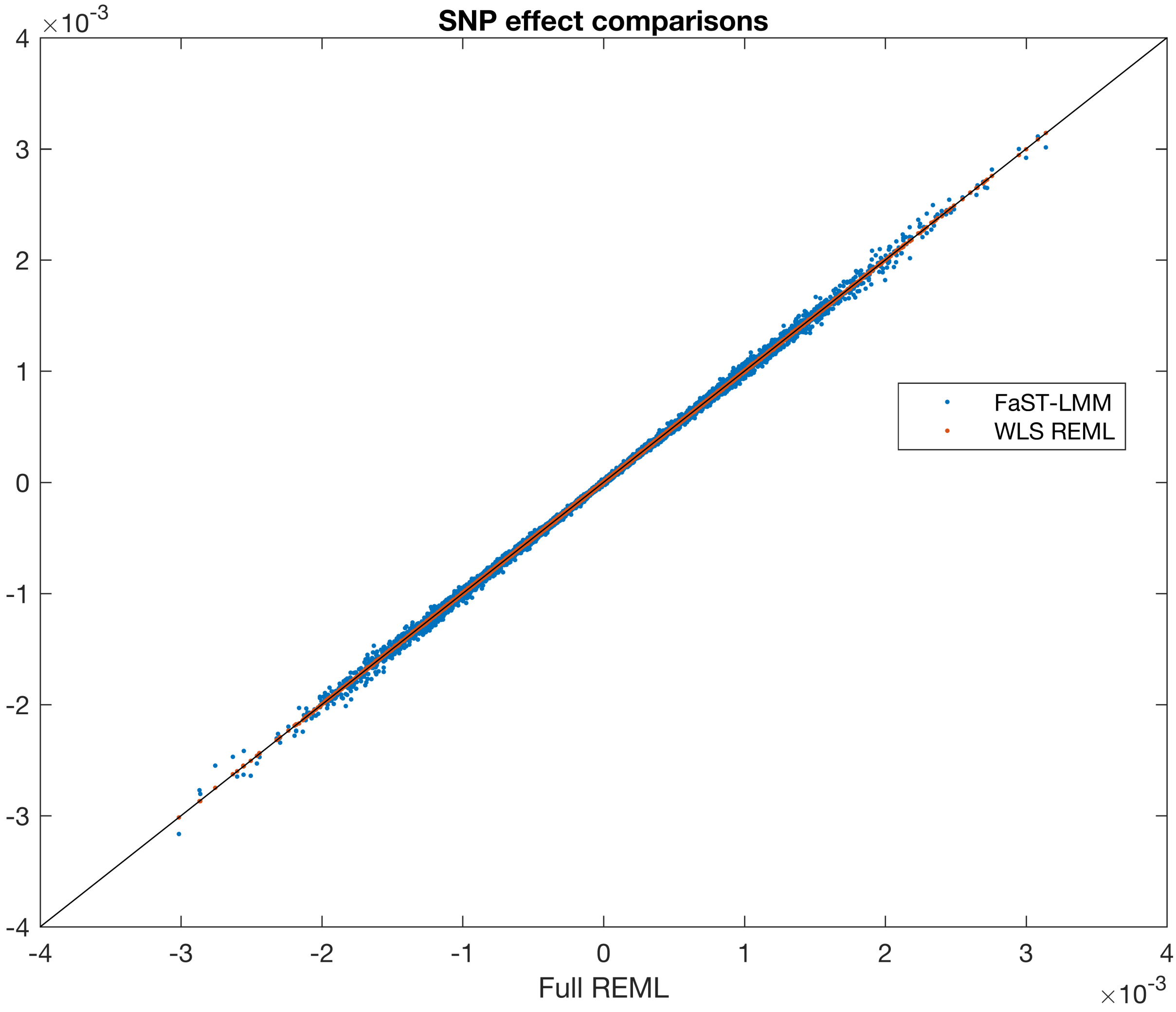

Supplementary Figure 7: Simulation 4, comparing fixed effect estimation bias between FaST-LMM and the simplified REML function. Each point represents a simulated SNP bias where 6000 SNPs with MAF range (0.05,0.5) are simulated. The x-axis shows parameter estimates bias (β_1) from Full REML and y-axis represents parameter estimates bias over 5000 phenotype realisations using FaST-LMM (blue points) and WLS-REML (red points).

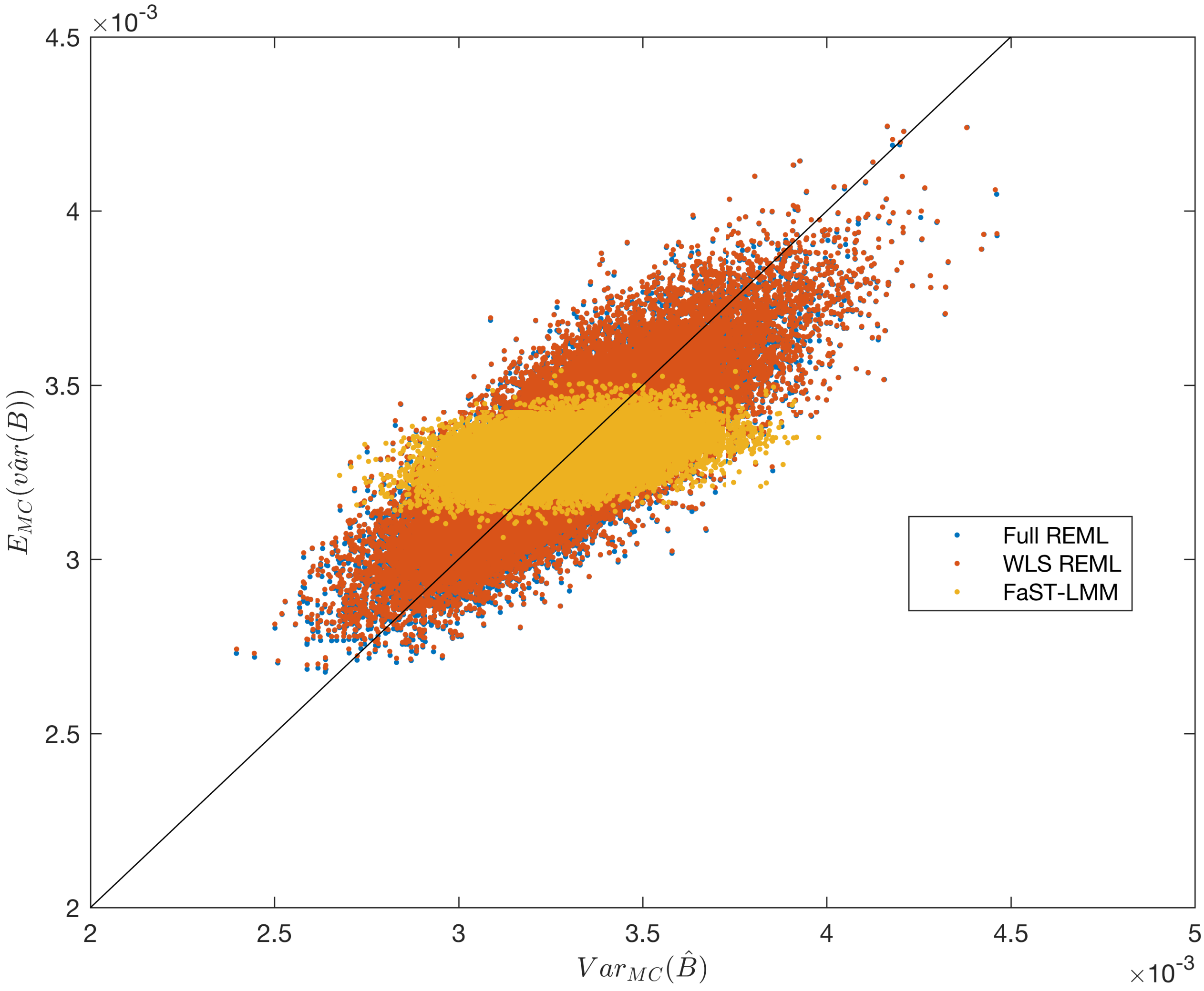

Supplementary Figure 8: Simulation 4, comparing the Monte Carlo (x-axis) and method-estimated (y-axis) variance of the fixed effect estimator, for FaST-LMM and the simplified REML function. 60,000 SNPs for 300 unrelated individuals with minor allele frequency between $(0.05, 0.5)$ were simulated. 6000 null markers ($\beta_1 = 0$) were used for comparison in 5000 realisations for each marker. In these simulations 54,000 markers were used to induce different level of heritability under the additive model $h^2_{\mathrm{A}} = 0.1, 0.2, 0.4, 0.6\&0.8$ and $\sigma^2_p = 1$ where markers are standardised to have mean zero and unit variance. There are 6000 points, one for each simulated SNP where the Monte Carlo estimate of variance is over the 5000 $\hat{\beta}_1$; each method's produces one $\widehat{\mathrm{Var}}(\hat{\beta}_1)$ for each realization, which are averaged to obtain the y-axis value. (See the **??** section for more details)

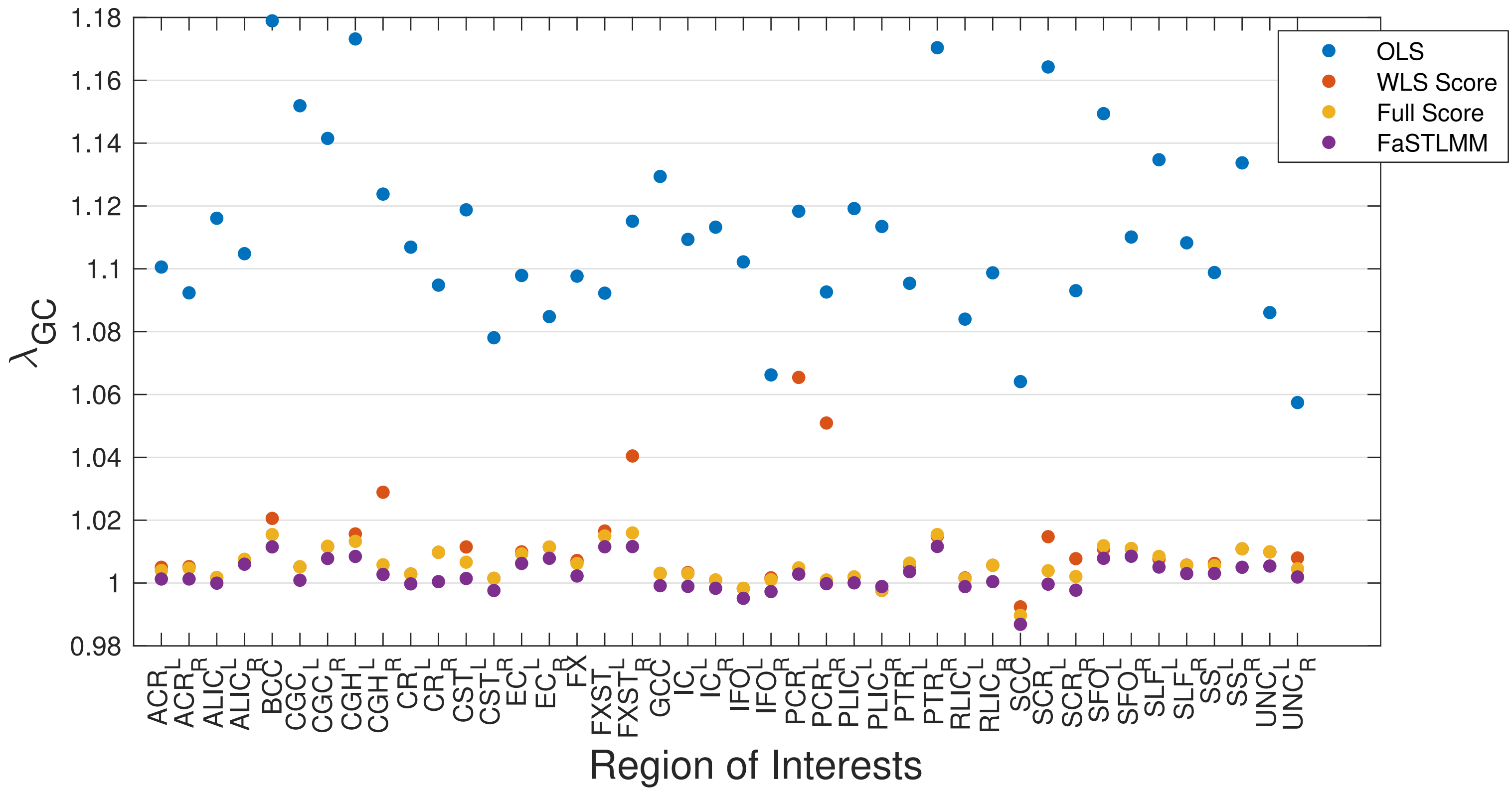

Supplementary Figure 9: Real data analysis, comparing the genomic control values of the score test based on the simplified REML function using either fully converged random effect estimator (Fully Score, yellow dots) or the WLS-REML random effect estimator (WLS Score, red dots), FaSTLMM (purple dots) with the linear regression with MDS as nuisance fixed effects (OLS, blue dots) for 42 ROIs in the CEU sample. Our proposed method consistently gives smaller genomic factor regardless of random effect estimation method and close to FaST-LMM.

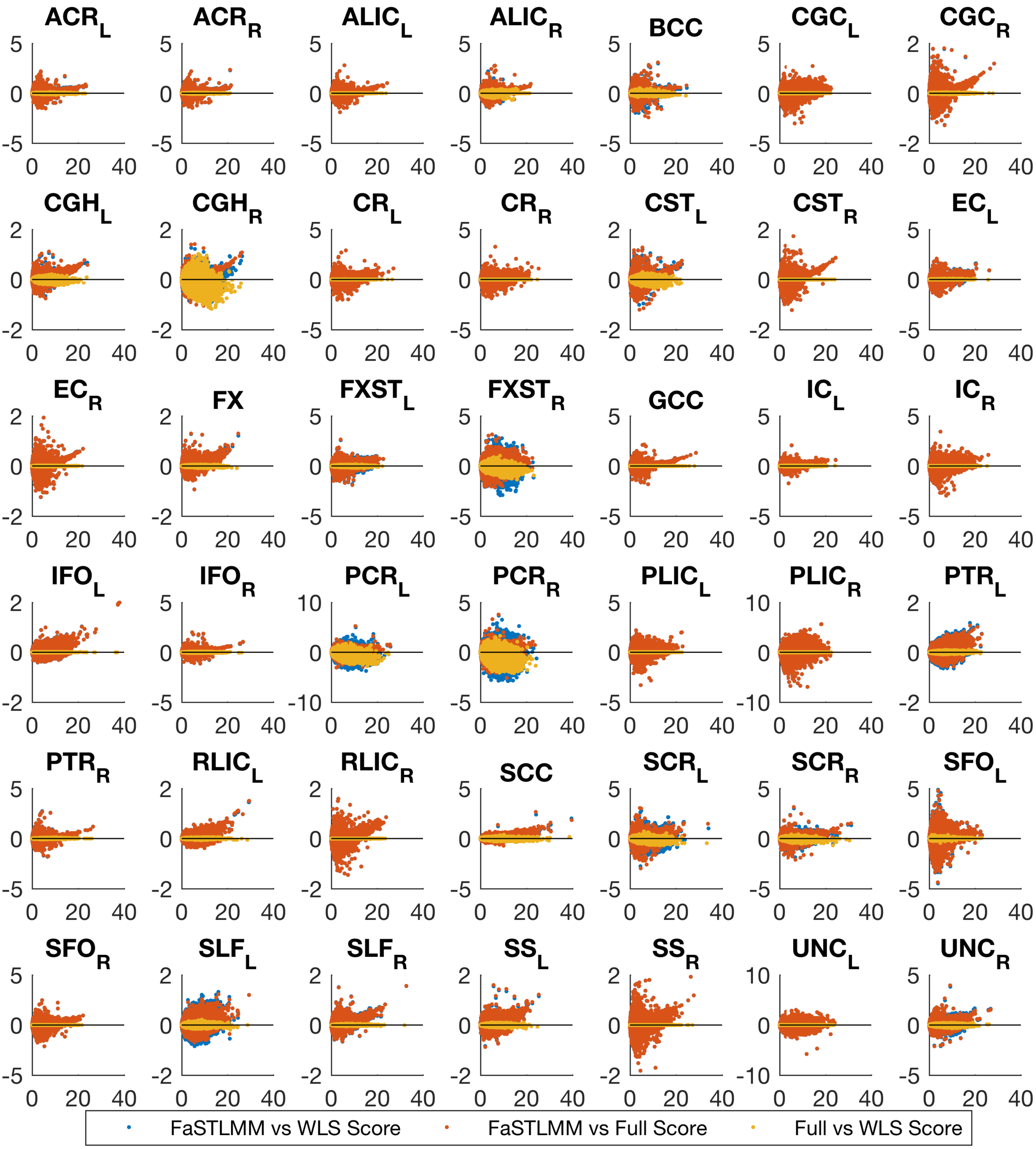

Supplementary Figure 10: Real data analysis, Bland-Altman plot for comparing values of FaST-LMM and the score test for association testing ($H_0 : \beta_1 = 0$) using non-iterative and fully converged random effect estimators. Each plot represents a ROI where x-axis shows average statistics and y-axis represents differences. The score tests are almost identical and slightly less powerful than FaST-LMM.

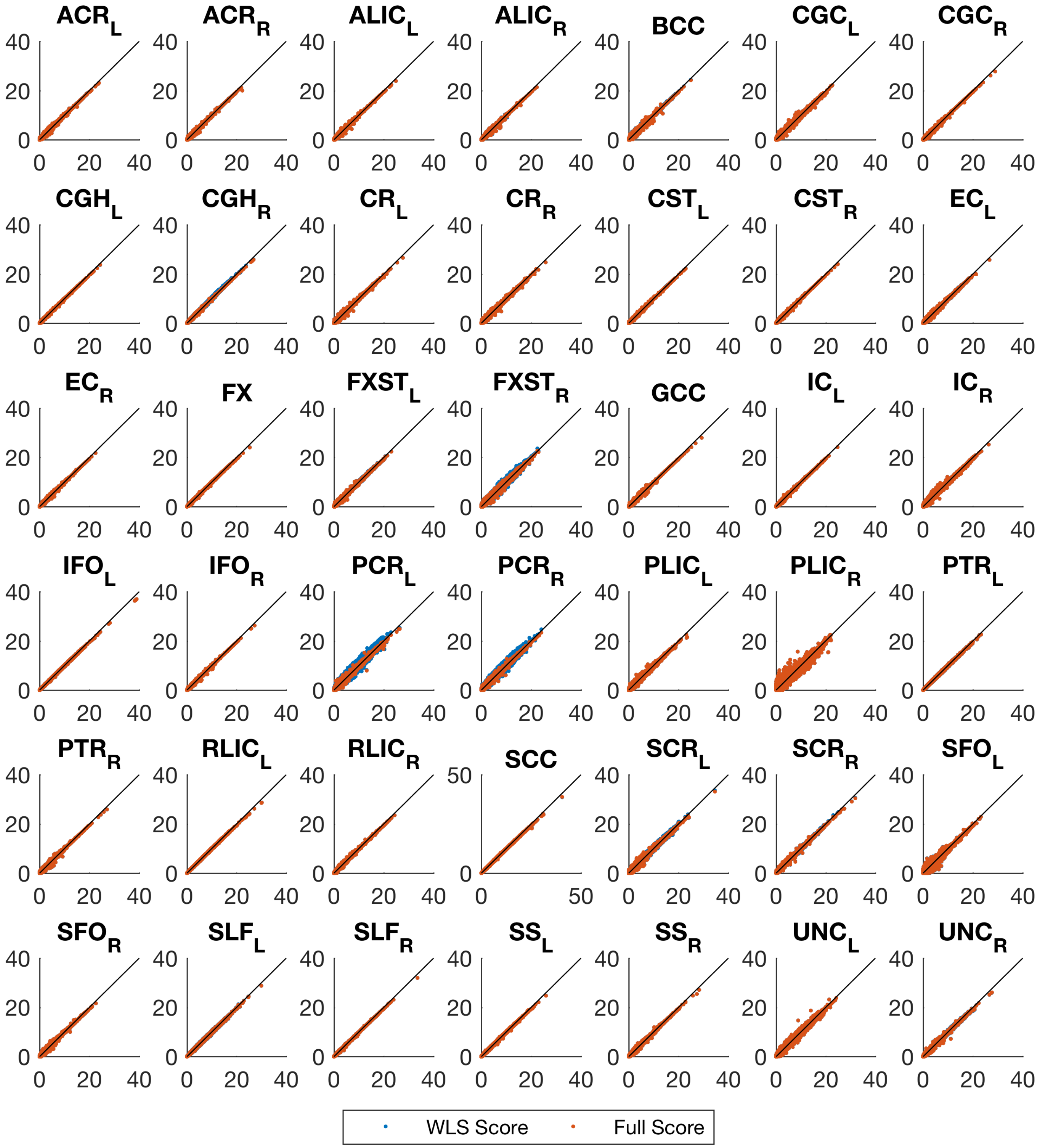

Supplementary Figure 11: Real data analysis, comparing values of the score test for association testing ($H_0 : \beta_1 = 0$) using non-iterative and fully converged random effect estimators. and Fast-LMM Each plot represents a ROI where x-axis shows FaST-LMM LRT values score test using estimator and y-axis represents score test using either WLS-REML or the fully converged random effect estimator. The score tests are almost identical and slightly less powerful than FaST-LMM.

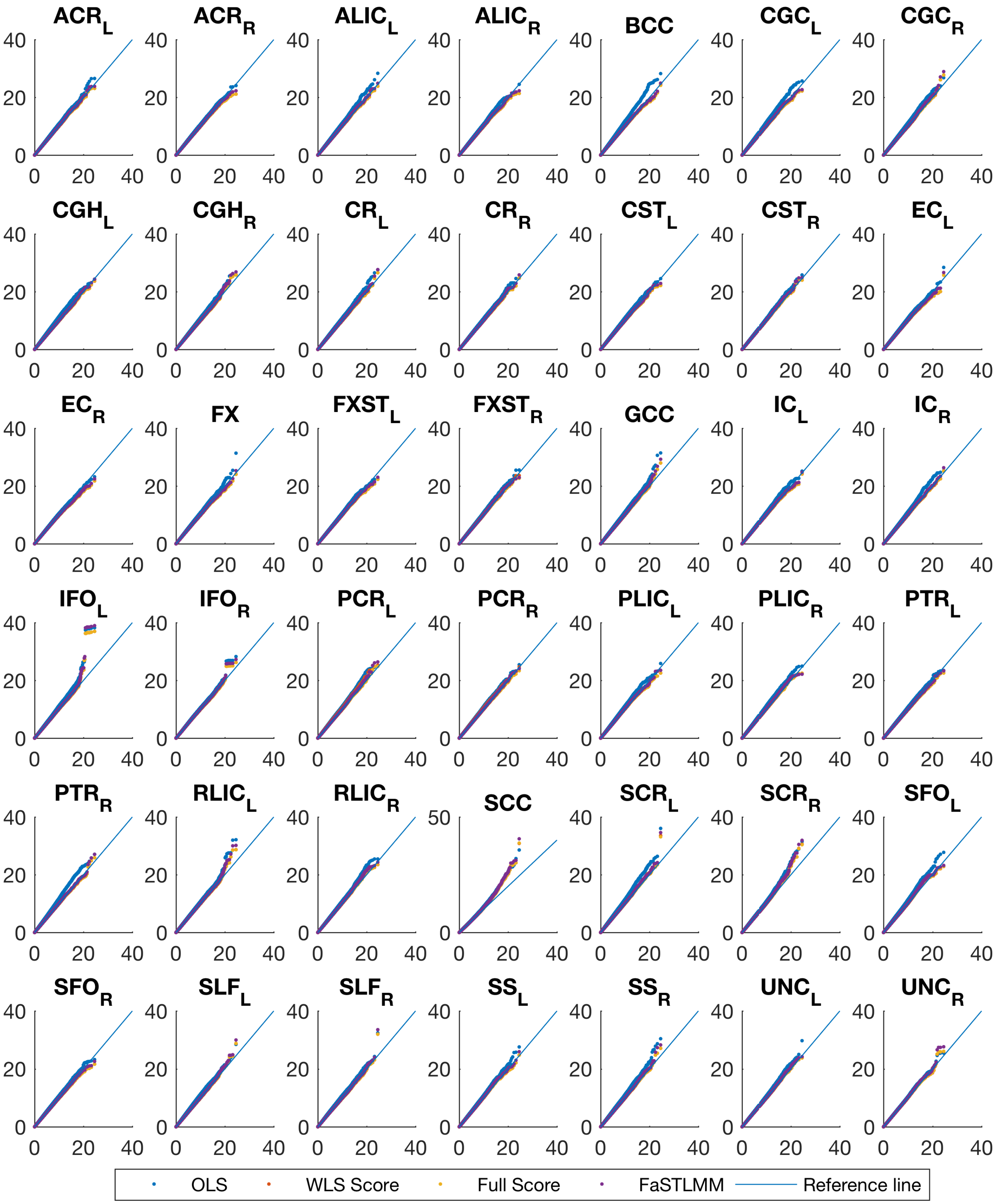

Supplementary Figure 12: Real data analysis, QQ plot for comparing FaST-LMM and the score test based on the simplified REML function using the WLS-REML random effect estimator with the linear regression with MDS as nuisance fixed effects. Each plot corresponds to different ROIs. These plots show either an identical distribution or slightly larger values for the OLS approach. However the OLS approach has poor genomic control (Figure 9).

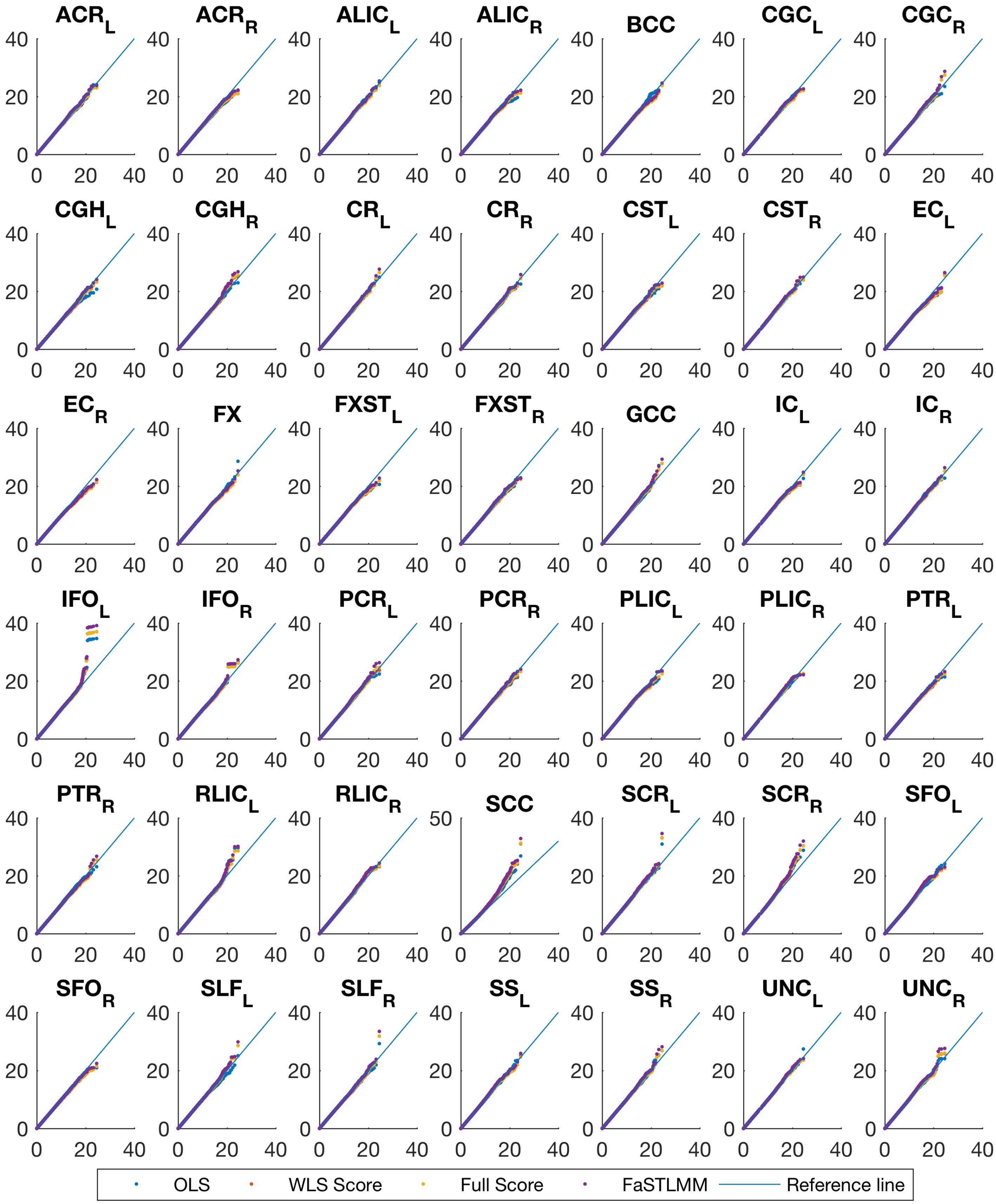

Supplementary Figure 13: Real data analysis, QQ plot for comparing the adjusted association statistics for genomic control values. Each plot corresponds to different ROIs. These plots show after adjustment we get essentially identical results for the score test based on the simplified REML function using the WELS REML random effect estimator and the OLS approach.

References

David C. Glahn, Paul M. Thompson, and John Blangero. Neuroimaging endophenotypes: Strategies for finding genes influencing brain structure and function. *Human Brain Mapping*, 28(6): 488–501, 007. doi: 10.1002/hbm.20401.

Derrek P Hibar, Jason L Stein, Miguel E Renteria, Alejandro Arias-Vasquez, Sylvane Desrivières, Neda Jahanshad, Roberto Toro, Katharina Wittfeld, Lucija Abramovic, Micael Andersson, Paul M Thompson, and Sarah E. Medland. Common genetic variants influence human subcortical brain structures. *Nature*, 520(7546):224–229, 04 2015. doi: http://dx.doi.org/10.1038/nature14101.

Christoph Lippert, Jennifer Listgarten, Ying Liu, Carl M. Kadie, Robert I. Davidson, and David Heckerman. FaST linear mixed models for genome-wide association studies. *Nature Methods*, 8 (10):833–837, 2011. doi: 10.1038/nmeth.1681.

Jiska S. Peper, Rachel M. Brouwer, Dorret I. Boomsma, Ren S. Kahn, and Hilleke E. Hulshoff Pol. Genetic influences on human brain structure: A review of brain imaging studies in twins. *Human Brain Mapping*, 28(6):464–473, 2007. doi: 10.1002/hbm.20398.

Jason L Stein, Sarah E Medland, Alejandro Arias Vasquez, Derrek P Hibar, and Enhancing Neuro Imaging Genetics through Meta-Analysis Consortium et al. Thompson P. M. Identification of common variants associated with human hippocampal and intracranial volumes. *Nat Genet*, 44 (5):552–561, 05 2012. doi: 10.1038/ng.2250.

Christian Widmer, Christoph Lippert, Omer Weissbrod, Nicolo Fusi, Carl Kadie, Robert Davidson, Jennifer Listgarten, and David Heckerman. Further improvements to linear mixed models for genome-wide association studies. *Scientific reports*, 4:6874, 2014. doi: 10.1038/srep06874.
